# Towards an ICF-based self-report questionnaire for people with skeletal dysplasia to study health, functioning, disability and accessibility

**DOI:** 10.1186/s13023-021-01857-7

**Published:** 2021-05-22

**Authors:** Heidi Anttila, Susanna Tallqvist, Minna Muñoz, Sanna Leppäjoki-Tiistola, Outi Mäkitie, Sinikka Hiekkala

**Affiliations:** 1 Public Health and Welfare Department, Knowledge Management and Co-Creation Unit, Finnish Institute for Health and Welfare, Mannerheimintie 166, 01270 Helsinki, Finland; 2 University of Helsinki, Yliopistonkatu 3, 00014 Helsinki, Finland; 3 Validia Ltd, Validia Rehabilitation, Nordenskiöldinkatu 18 B, 00250 Helsinki, Finland; 4 Lyhytkasvuiset – Kortväxta ry (Finnish Association for People with Restricted Growth and for Their Families), PO Box 14, 02601 Espoo, Finland; 5 Finnish Association of People With Physical Disabilities, Mannerheimintie 107, 00280 Helsinki, Finland; 6 Children’s Hospital, University of Helsinki and Helsinki University Hospital, P.O. Box 63, 00014 Helsinki, Finland; 7 Folkhälsan Institute of Genetics, Helsinki, Finland

**Keywords:** Functioning, Disability, Environmental factors, Self-report, Questionnaire design, Content validity, Skeletal dysplasia, Short stature, Rare disease

## Abstract

**Background:**

Little is known about the spectrum of everyday challenges that people with skeletal dysplasia face because of their health and functioning. We aimed to identify factors related to health, functioning and disability in people with skeletal dysplasia, and their challenges with accessibility in order to form a self-reported questionnaire for national data collection. The comprehensive musculoskeletal post-acute core set of the International Classification of Functioning, Disability and Health (ICF) was used as a framework.

**Methods:**

An iterative, participatory and qualitative process was used to formulate a questionnaire. Items were searched from Patient-Reported Outcomes Measurement Information System and from other self-report instruments, additional items were formulated using ICF linking rules. Expert panels from the target population assessed the face and content validity in thematic interviews.

**Results:**

The questionnaire demonstrated its relevance, comprehensiveness and feasibility for people with skeletal dysplasia. The ICF linkages showed the contents’ correspondence to the construct. Expert panels added 15 categories and one on chapter level to the core set and confirmed content validity. The final survey covers 86 ICF categories and 173 ICF-linked items that were grouped to 33 questions.

**Conclusions:**

The content of the questionnaire proved to be sufficiently valid for people with skeletal dysplasia. It can be used to explore their health, functioning, disability and accessibility to develop care and rehabilitation policies, to plan services and to provide information to various parties involved.

**Supplementary Information:**

The online version contains supplementary material available at 10.1186/s13023-021-01857-7.

## Introduction

Individuals with short stature experience several challenges in the accessibility in society although they have the same rights as people with normal height [41]. Health and functioning play a major role in enabling them to participate in daily activities, social events and society. Short stature is a predominant feature in several rare conditions classified as skeletal dysplasia. These disorders affect the musculoskeletal system already prenatally and during the years of growth. The affected children differ in height from their peers and the attained adult height is less than 140–150 cm. [24]).

There are numerous different medical reasons for short stature, including more than 400 hereditary diseases of the skeletal system (skeletal dysplasias), chromosomal aberrations, hormone deficiencies and developmental disorders [24]. Classification solely by categorical diagnosis does not provide sufficient information about the impact of the condition on individuals’ lives and “lived experience”. The few earlier small studies have reported conflicting results [35] on selected aspects of functioning in specific skeletal dysplasias, such as health-related quality of life [1, 42], pain and self-care, mobility and participation [18], as well as income, education and partnerships [15]. Planning of care, rehabilitation and social services, however, requires detailed and accurate knowledge about individual life situations. Recent legislative developments in Europe and United States and high public administration bodies support efforts to include patients' reports of health experience in order to engage in shared decision-making, prioritize the focus of treatment and services and to evaluate treatment outcomes [5, 10]. Therefore, in health-related studies in people with short stature, it is of great importance to include assessment of functioning, disability and accessibility.

The World Health Organisation (WHO) has developed the International Classification for Functioning, Disability and Health (ICF), among others, for describing functioning in relation to health condition [46]. The ICF is part of the WHO’s family of the international classifications, developed to provide a comprehensive and universally accepted framework to understand the lived experience of health in individuals as well as in populations. The concept of functioning is introduced explicitly in the biopsychosocial model that forms the ICF framework [28]. According to this model, the level of a person’s individual functioning is the outcome of a dynamic and complex interaction between the health condition, body functions and structures (physiological functions and anatomical parts of the body system), activities (execution of a task) and participation (involvement in life situations), personal factors (features intrinsic to an individual) and environmental factors (physical, social and attitudinal environment), which can be facilitators or barriers. The interaction between these components is dynamic and bidirectional, changes in one component may influence one or more of the other components.

In addition to the framework, the ICF has over 1600 categories providing an exhaustive classification of an individual’s functioning, suitable for all people. Core sets were made to help focus on the most important factors of certain health statuses [34]. The available 35 health-condition and context specific ICF core sets cover most burdensome health conditions [32], while the ICF Rehabilitation set (also called as Generic-30) is recommended for broad clinical and rehabilitation contexts [26] and the short ICF Generic-7 set to be used always [6]. Tools for assessment of functioning can be derived from core sets suitable for target population, validated and implemented to ensure that the ICF framework is applied [27, 33]. Though the ICF has been accepted in all WHO member states, it has been mostly applied in neurological, musculoskeletal and work-related contexts [21]. We found no ICF-based tool for people with short stature due to skeletal dysplasia.

## Methods

### Aim, design and setting

In this study we aimed to formulate a self-report questionnaire using the ICF to study health, functioning, disability and accessibility in people with short stature due to skeletal dysplasia. The questionnaire was needed for a national study to explore the lived experience of functioning, disability and health of people with short stature, as well as their challenges concerning accessibility and equality, to guide in planning their health care and rehabilitation services on the national level. In our previous study [17] the questionnaire was applied to 80 people with skeletal dysplasia, and the scoring method and the results were described. In the present study the questionnaire’s content validity was evaluated using the following research questions: Are all items relevant for the intended purpose of the questionnaire? Are all items relevant for people with skeletal dysplasia with respect to age, gender and disease characteristics? Do all items refer to relevant aspects of the construct to be measured?

### Target population

We considered short stature, disproportion, deformities and joint restrictions to be the major features affecting functioning in skeletal dysplasias. The target population was specified as people with short stature due to one of the three most common skeletal dysplasias in Finland: diastrophic dysplasia, achondroplasia and cartilage-hair hypoplasia. Their common clinical features are musculoskeletal problems and severe short stature (Table 1). Diastrophic dysplasia is one of the most severe skeletal dysplasias with significant deformities and joint contractures while achondroplasia is the most common skeletal dysplasia world-wide. People with short stature were recruited by announcements and personal contacts.

**Table 1:** Characteristics of the three most common congenital skeletal dysplasias in Finland

Health condition	Clinical features
Diastrophic dysplasia (autosomal recessive inheritance) OMIM #222,600	Disproportionate short stature with short arms and legs, scoliosis, joint deformities and contractures, and foot deformities
	Normal mental development and life expectancy
	Progressive degenerative changes of the articular cartilage and severe joint deformities often require hip and knee arthroplasties at an early age
	Adult height 130–140 cm
	More common in Finland than in any other country
Achondroplasia (autosomal dominant inheritance) OMIM #100,800	Characteristic appearance of disproportionate short stature with short limbs and long spine
	Other complications include e.g. delayed motor milestones and leg deformities in childhood; spinal stenosis, pain, and complications with aging
	Normal cognition and overall physical development and are productive and independent adults
	Adult height 120–135 cm
	The most common skeletal dysplasia worldwide
Cartilage-hair hypoplasia (autosomal recessive inheritance) OMIM #250,250	A highly pleiotropic disorder with many features involving various extra-skeletal organ systems
	Disproportionate stature with short limbs, normal joint function, sparse hair, variable immunodeficiency and predisposition to malignancies
	Normal intelligence and developmental milestones
	Adult height 104–149 cm
	More common in Finland than in any other country

### Construct

The construct to be evaluated was defined as health, functioning, disability and accessibility. The term “health” was defined as diagnosis, as described in the ICD-10 (International Statistical Classification of Diseases and Related Health Problems) [47] or as health problems described by ICF body functions (BF). The latter terms were defined using the ICF as a framework. “Functioning” and “disability” were described by using categories from the ICF body functions (BF), body structures (BS), activities and participation (A&P) domains and ”accessibility” by facilitators or barriers in the environmental factors (EF) domain. Because the skeletal dysplasia can be classified to rare musculoskeletal diseases, we chose the comprehensive musculoskeletal post-acute ICF core set as an initial set of “what to measure” [31]. This core set includes 70 ICF categories (23 in BF, 7 in BS, 22 A&P, and 18 in EF). It covers all 7 categories in the ICF Generic set and 36 of the 42 categories of the ICF Rehabilitation set with environmental factors.

### Questionnaire development

**Fig. 1:**
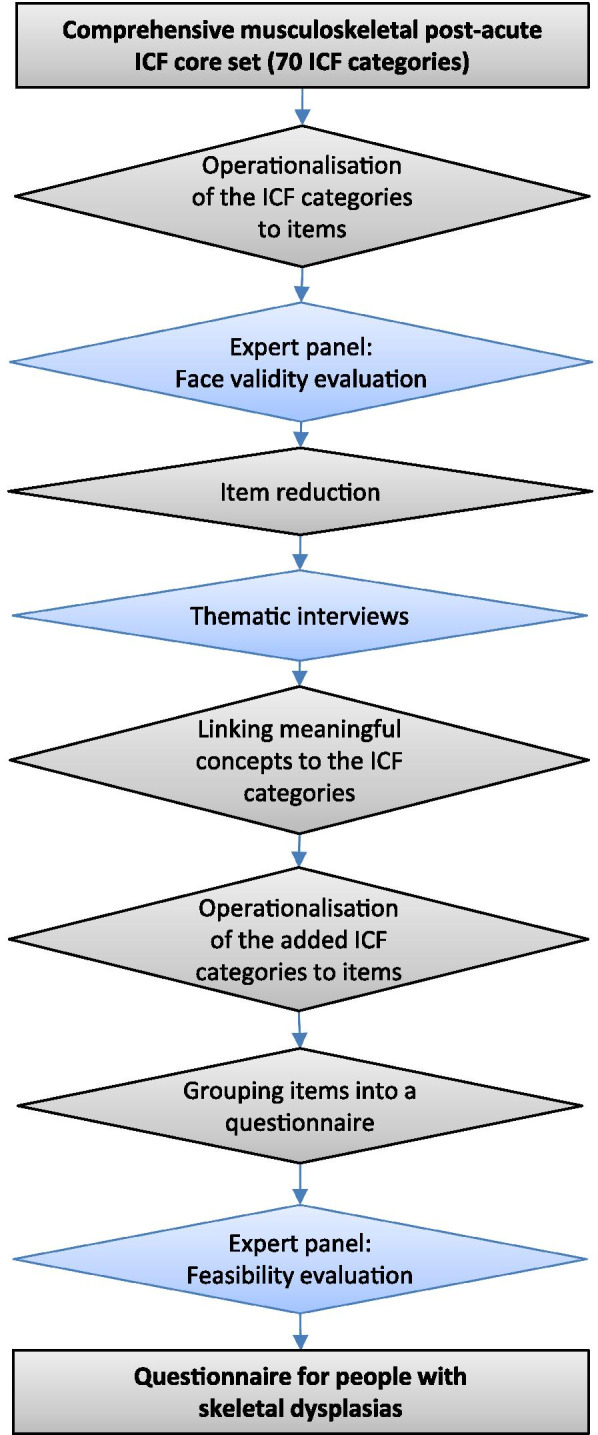
The process of the questionnaire development for people with skeletal dysplasias

The questionnaire development (Fig. 1) was carried out between February and August 2016. The research group (authors) has wide experience of medical, health, and social sciences and the target group. To ensure participatory approach throughout the study, an expert panel (n = 4), representing different skeletal dysplasia diagnoses (Table 1), was formed to assess face validity, feasibility and acceptance of the survey.

Requirements for items were that an item (a question or a phrase) should be relevant and acceptable for subjects with short stature due to skeletal dysplasia, capture the person’s subjective valuation of symptoms or functioning i.e. patient reported outcome (PRO), be in 1st person, be simple to understand and easy to respond, be publicly available, and should be able to be linked to one of the preselected ICF categories using the updated ICF linking rules [7]. A requirement for the final questionnaire was that completion time should not be too long and the survey should not include overlapping questions. Thus, the number of items should be adjusted so as to reach completion time of 15–20 min.

We sought items from validated self-report instruments. The Patient-Reported Outcomes Measurement Information System (PROMIS®) item banks have been developed by comprehensive literature searches of existing measures, using appropriate cognitive testing and contemporary psychometric methods [4]. The PROMIS has demonstrated rapid, accurate measurement and clinical validity across a range of chronic conditions [9]. This system provides extensively developed self-report items for assessing physical, mental, and social health in 25 item banks and short-forms derived from these banks. The items are short and easy to understand [11], have short recall time (7 days) except physical function items [8], and five-point answering options [25]. Tucker et al. [38] demonstrated harmonization and synergy between the PROMIS and the ICF conceptual frameworks. The mapping of 1007 items from PROMIS adult item banks to ICF code descriptors shows that the PROMIS items provide a basis for majority of concepts in the ICF BF and A&P categories [39].

To identify items for ICF EF categories we utilized several sources. Recent reviews [20, 29] identified self-report measures on how environmental barriers or facilitators impact participation for people with disabilities: Craig Hospital Inventory of Environmental Factors (CHIEF) [44] and Measure of the Quality of the Environment (MQE) [14]. The 25 CHIEF items ask “In the past 12 months, how often has [an EF] been a problem for you”, and then “when this problem occurs has it been a big problem or a little problem”. The MQE asks “While taking into consideration your abilities and personal limits, indicate to what extent the following situations or factors generally influence your daily life” for 109 EF listed. National Finsote Survey covers some EF and is implemented throughout Finland to monitor the welfare and health of their residents [13]. As the CHIEF and MQE were not available in Finnish, we utilized their items as a basis to formulate new items. Lastly, if no item for the ICF categories could be identified from existing sources, the research group phrased a new item using the ICF definition and existing PROMIS item formats (stems, responses, tense and person [11].

### Assessment of face and content validity

The expert panel was asked to do an overall view of the items (i.e. face validity) [12] and to accept “suitable” items, reject “redundant” items and provide comments for each item. Thereafter, item reduction aimed to come down to a reasonably low number of items to reach the preset completion time limit. Decision rules for keeping items were: (1) all of the four persons appraised the item as relevant and, (2) the item was in the PROMIS short form, as these items have best discriminative power [30].

We then searched target group input to assess whether the content of the questionnaire corresponds with the construct and its’ relevance and comprehensiveness (i.e. content validation) [37]. This was done by structured, thematic interviews to understand how people with short stature experience functioning and accessibility and equality, and their review of the draft questionnaire. Fourteen signed informed consent (2 men, 9 women and 3 children aged 9–12 years with their parents), representing all three diagnoses and geographically different areas in Finland. Themes in the interview were functioning, accessibility and equality. The participants could tell about the above-mentioned themes in a preferable order. If needed, these themes were clarified to the participants by questions that were description objects from the ICF framework. In the end of interview the interviewer gave the participants the draft questionnaire and asked for its understandability and relevance. Thereafter, the participants were asked to accept or delete items, and provide comments, if needed, for each item.

The interviews lasted from 46 min to 1 h and 26 min. Exact transcriptions of the thematic interviews were performed. The analysis was first based on the data and then linked to the theory i.e. the ICF framework [23, 40]. From the transcriptions we identified meaningful concepts about functioning (linking units) and mapped them to the ICF using the ICF linking rules [7]. The resulting ICF categories were compared to the 70 categories of comprehensive musculoskeletal post-acute core set.

## Results

### Relevance of the items for the questionnaire’s planned use

From the available item banks and questionnaires, we identified altogether 522 possible relevant items for a national questionnaire. Altogether, these items covered 37 categories of the chosen ICF core set (0 BS, 5 BF, 19 A&P, 13 EF). There were 452 possible PROMIS items for 28 core set categories. The number of items per category varied from 1 to 108, for example, 108 items for energy and drive functions (b130), 18 for fine hand use (d440), and only one for transferring oneself (d420). The PROMIS covered 19 of the 22 A&P and 5 of the 23 BF categories. Moreover, from the PROMIS we could identify a few items for EF, but on chapter level (e3, e4) only, as in those items one cannot say who gives support or whose attitude is in question. However, we accepted them to cover the ICF categories e355 and e410, e430, e440, respectively.

From the Finsote survey we identified two items about social life and 26 services. These items were modified to address 1st person, and we added transportation services. From CHIEF and MQE we identified 25 items that were used as basis for item development, covering 10 of the 18 EF categories. The remaining EF categories and the many BF categories, not covered by any available items, were operationalized to items based on the ICF category definition. The EF items asked about accessibility at home and in other buildings and public places, as well as attitudes. The BF items inherently ask about health conditions or functions. The core set categories for weight (b530) was considered as a background variable, to which we added items outside the core set about age, height, cordage, gender, and life situation. The 7 BS categories were regarded non-relevant as self-report items, as these structural impairments were covered in the diagnoses (see Table 1) and were thus not operationalized as single items.

### Relevance of the items for people with skeletal dysplasia

The initial ICF core set related list included 531 items, most accepted by the expert panel as having good face validity. After item reduction, 161 items were kept and grouped to 117 questions in a draft questionnaire. Based on the comments from thematic interviews, 31 items were discarded, 9 modified and 8 added. The results from the thematic interviews brought more qualitative insight of the functioning, disability and accessibility experience by people with skeletal dysplasia. The themes originating from content analysis comprising of nine classes could be linked to all ICF domains: daily barriers (ICF BF and BS); work and leisure time, challenges in mobility and daily activities (ICF A&P); facilitators for mobility and activities, social support, attitudinal environment, services, physical environment as a barrier or facilitator (ICF EF); and attitude towards oneself (ICF personal factors). The qualitative results are published elsewhere [16]. For the questionnaire development, the transcriptions provided details of the functioning, disability and accessibility changes that these people meet in their everyday life. In the transcriptions, there were 46 subcategories regarding functioning and environmental factors. Their linkages into the ICF categories are shown in Table 2. The linking confirmed the selection of several core set categories and yielded additional categories.

**Table 2:** Meaningful concepts from interviewed people with skeletal dysplasias (n = 14) linked to the ICF categories

Count	Meaningful concepts on functioning or accessibility	ICF code	ICF title	Decision
1	Mental functioning	b130*	Energy and drive functions (G)	Keep
2	Sleeping	b134*	Sleep functions	Keep
3	Pain	b280*	Pain (G)	Keep
4	Allergies, infections	b435*	Immunological system functions	Keep
5	Stiff joints	b710*	Mobility of joint functions	Keep
6	Loose joints	b715*	Stability of joint functions	Keep
7	Muscle strength	b730*	Muscle power functions	Keep
8	Significance of a skill	d155*	Acquiring skills	Keep
9	Basic movements (turning oneself, getting up sitting from lying, getting lying down on stomach, moving on ones buttock)	d410*	Changing basic body position	Keep
10	Using hands	d445*	Hand and arm use	Keep
11	Walking	d450*	Walking (G)	Keep
12	Running, climbing stairs	d455	Moving around	Add
13	Moving with public vehicles	d470	Using transportation	Add
14	Personal hygiene, taking care of beauty	d520*	Caring for body parts	Keep
15	Toileting, peeing and cannot wipe oneself	d530*	Toileting	Keep
16	Dressing	d540*	Dressing	Keep
17	Eating	d550*	Eating	Keep
18	Difficulty of accessing services	d620	Acquisition of goods and services	Add
19	Preparing meals	d630	Preparing meals	Add
20	Doing housework e.g. washing clothes	d640	Doing housework	Add
21	Friends	d750	Informal social relationships	Add
22	Work as a resource, doing work, having a career	d850	Remunerative employment	Add
23	Hobbies (sports, games, circus, playing music, movies etc.)	d920	Recreation and leisure	Add
24	Experiencing oneself as a person with disability	d940	Human rights	Add
25	Small assistive technologies, special shoes	e115*	Products and technology for personal use in daily living	Keep
26	Assistive technologies for moving (car, electric wheelchair or scooter)	e120*	Products and technology for personal indoor and outdoor mobility and transportation	Keep, but consider car
27	Public vehicles	e1200	General products and technology for personal indoor and outdoor mobility and transportation	Consider in e575
28	Changes to a car	e1201	Assistive products and technology for personal indoor and outdoor mobility and transportation	Consider in e120
29	Accessibility at public areas (study places, bank automates, banks, shops, high desks, stairs, heavy doors)	e150*	Design, construction and building products and technology of buildings for public use	Keep
30	Accessibility at home, changes to one’s apartment (socles, additional taps, heightened floor at balcony, handles and grips, lowered washbasin, power sockets, personal bath chair and furniture)	e155	Design, construction and building products and technology of buildings for private use	Add
31	Accessibility at home entrance (lowered door handles, electric lock, door pumps)	e1550	Design, construction and building products and technology for entering and exiting of buildings for private use	Consider in e155
32	Seasonal changes	e245	Time-related changes	Add
33	Support from family	e310*	Immediate family	Keep
34	Support from friends and peers	e320*	Friends	Keep
35	Support from personal assistant	e340*	Personal care providers and personal assistants	Keep
36	Other people offer help without asking	e345	Strangers	Add
37	Attitudes in public services	e445	Individual attitudes of strangers	Add
38	Attitudes of health workers	e450*	Individual attitudes of health professionals	Keep
39	Inequal access to services	e455	Individual attitudes of other professionals	Add
40	Other people's attitudes, discrimination	e460	Societal attitudes	Add
41	Public transportation services	e540	Transportation services, systems and policies	Consider in e575
42	Support from society (disability services and benefits)	e575	General social support services, systems and policies	Keep
43	Access to assistive products and transportation services	e580*	Health services, systems and policies	Keep
44	Other challenges (lymphatic drainage in the brain)	s110	Structure of brain	Not include
45	Joint detrition, bone fractures, accidents	s7	Structures related to movements	Add
46	Bone displacements (correction surgery)	s770	Additional musculoskeletal structures related to movement	Consider in s7

^*^Category belongs to the comprehensive musculoskeletal ICF core set

Based on the content validation, no core set category was deleted, but we added 15 second level ICF categories (9 A&P and 6 EF), which were not included in the comprehensive musculoskeletal post-acute ICF core set. Moreover, the body structures that were first considered only as diagnoses were added at chapter level (s7). To operationalize the new categories, we added 33 items: 11 from PROMIS and 22 newly formulated items, including the three items for BS. Additional file 1 provides an overview of the number of items and their sources for each ICF category of the comprehensive post-acute musculoskeletal core set and the additional categories.

### Feasibility of the questionnaire

A questionnaire was formulated, using the Webropol software. As the items could be grouped by similar answering options, the items did not follow the order of the ICF categories. Instead, the items were grouped to 32 questions under 10 lay language titles: sociodemographic factors; health and body functions; home, assistive products and vehicles; mobility; daily activities; mental welfare; pain; social relationships; work and leisure time; and social and health services. The expert panel tested the feasibility of the questionnaire and provided qualitative insights on fluency, understandability of the newly formulated items, as well as ordering of items and suitability of item grouping. Changes were implemented, largest change was splitting one item for d470 into two i.e. transportation with and without luggage. One open question was added. The questionnaire was proof-read and finalized with 34 questions, consisting of 173 ICF-linked items (Additional file 1) and 6 sociodemographic items (contact information, age, length, cordage, gender, and life situation), and one open question. All the four individuals with skeletal dysplasia accepted the final questionnaire and completed it. The completion time was 15–20 min.

### Correspondence to the construct

The use of the ICF linking rules demonstrated, to what ICF domains and categories the items in the questionnaire belong. All the additional items address health, functioning or accessibility and thus belong to the construct. Thus, the final number of ICF categories was 86, comprising of 25 categories describing the construct of health (8 BS and 17 BF), 35 functioning and disability (6 BF, 31 A&P), and 24 accessibility (ICF EF). Table 3 illustrates the correspondence of the lay titles and items in the final questionnaire to the ICF domains, the themes identified from thematic interviews, and the construct terms.

**Table 3:** The correspondence of the questionnaire to the ICF domains, the identified themes and the construct

Titles (number of questions) in the questionnaire	Number of ICF-linked items (n = 173)	ICF domains in the questionnaire (number of categories, n = 86)	Themes identified in content analysis from thematic interviews	Construct
1. Background information (5)*	2	Body structures (7), Body functions (1)		Health
2. Health and body functions (6)	42	Body structures (1), Body functions (16)	Daily barriers (ICF BF and BS)	Health
3. Home, assistive products and vehicles (4)	5	Environmental factors (2)	Physical environment as a barrier or facilitator (ICF EF)	Accessibility
4. Mobility (3)	30	Body functions (2) activities and participation (10), environmental factors (3)	Challenges in mobility and daily activities (ICF A&P), Facilitators for mobility and activities (EF)	Functioning and disability, accessibility
5. Daily activities (2)	25	Activities and participation (14), environmental factors (3)	Challenges in mobility and daily activities (ICF A&P)	Functioning and disability, accessibility
6. Mental welfare (3)	13	Body functions (3), activities and participation (3)	Attitude towards oneself (ICF personal factors)	Functioning and disability,
7. Pain (3)	4	Body functions (1)		Functioning and disability
8. Social relationships (2)	16	Activities and participation (3), environmental factors (9)	Social support (EF), attitudinal environment (EF)	Functioning and disability
9. Work and leisure time (3)	6	Activities and participation (1), environmental factors (1)	Work and leisure time (A&P)	Functioning and disability, accessibility
10. Social and health services (2)	30	Environmental factors (6)	Services (EF)	Accessibility

^*^Only two of the background items were ICF-linked (weight and diagnosis)

## Discussion

In the present study, we describe how to operationalize an ICF core set to capture lived experience of persons with a rare disease, such as short stature due to skeletal dysplasia. Items were searched from state-of-art item banks, such as the PROMIS and other widely used questionnaires or surveys. Many items, particularly on EF had to be newly formulated in the Finnish language. People with skeletal dysplasia were consulted in all phases. As a result, a questionnaire was compiled in an iterative, participatory and qualitative process that demonstrated its preliminary relevance and feasibility for people with skeletal dysplasia.

Content validation is the most important step in questionnaire development [37]. We gave information about construct and situation for which the questionnaire was developed. The expert panel of individuals with skeletal dysplasia assessed the face validity of the questionnaire after which experts from the target population assessed whether the questionnaire content was relevant and comprehensive in a qualitative study. The correspondence to the construct was assessed by linking the items to the ICF categories.

Previously, the Quality of Life in Short Stature Youth instrument have been developed for people with short stature and used to study their health-related quality of life, including physical, social and emotional domains clinical contexts [3, 19]. The instrument targeted children and adolescents and their parents. In this study, however, we developed a survey for adults to be used in national context with wider content as guided by the comprehensive musculoskeletal post-acute ICF core set and the thematic interviews.

This study provides further evidence for the ICF core sets’ applicability in survey design (see also [36]) and the use of the ICF framework for content validation of a questionnaire (see also [43]). The selected ICF core set worked well as a basis for the survey as well as for the interview themes. Meanwhile, the WHO has published a supplementary section for functioning assessment in the ICD-11, to enable joint use of ICF with the ICD [45]. This chapter includes 47 functioning domains of high explanatory power. One benefit of having codes for each questionnaire item is to enable comparison. Our ICF-based questionnaire for people with short stature covers 29 of the 47 functioning domains of the ICD-11, but the ICD-11 lacks the further 58 categories that were meaningful for people with short stature.

There were many reasons for choosing PROMIS items to operationalize the ICF categories on functioning. The PROMIS tools are being adopted to assess a broad range of disease outcomes worldwide, as they enable a common metric for tracking outcomes across providers and medical systems [2]. By applying PROMIS items, the questionnaire has taken one step further for more standardized assessment of functioning across conditions. The used health domains of PROMIS measures included physical function, pain, fatigue, sleep disturbance, self-efficacy, satisfaction with social roles and activities, ability to participate in social roles and activities, and social support. The PROMIS short forms were translated into Finnish in a collaborative FinSCI study [36].

The PROMIS item banks provided both too many and too few items. To overcome excess of items for the same category, short forms of these item banks were considered, however, only single items could be utilized to reduce patient burden. Future studies should investigate the possibility to apply item banks via computer adapted testing (CAT). This would allow calculating results per health domain, and at the same time with reduced patient burden provide more exact results than the short forms [4]. Also, many new items needed to be formulated, particularly for BF and EF not available in PROMIS item banks. The health condition items covered BF well, but item development for EF was more cumbersome. The CHIEF and MQE were not available in Finnish, so we could only use them at issue level to help item formulation in Finnish.

Normal accessibility criteria are often of no use for people with short stature. Accessibility is not only question of reachability, but a matter of being able to use buildings and traffic systems, work or enjoy leisure time with or without technical aids, as other people. In addition to the existing items, the target group participants were able to inform in the interviews what and how to formulate items to ask about these important environmental factors. Moreover, representatives of the target group were important and active participants throughout the study; in study design, selection of the content, reviewing the analyses and confirming the results. They ensured that the final questionnaire became feasible and non-burdensome. All the new items were commented and accepted by the group of four individuals with short stature, however, these items should go under more rigorous cognitive testing in the future.

The study included a number of qualitative methods that were used iteratively and ensured that voices of people with short stature were heard. The thematic interviews provided important means to identify what is important about functioning and accessibility to people with short stature and to confirm the relevance of the survey content. The analyses were first conducted based on the findings, and then linked to the ICF. The linking proved to be clear, and there were no big discrepancies between the researchers. This revealed that the preselected categories from the comprehensive musculoskeletal post-acute ICF core set proved to be useful, but did not cover all relevant aspects. Fourteen new categories were added based on personal perspectives identified in qualitative interviews. The themes and issues remained the same across the participants, thus we consider that saturation was reached: new interviews did not reveal substantially new information. Studies have shown that saturation point can be reached in qualitative studies with 11 participants [22] and our study included 14 participants.

This study has number of limitations. The questionnaire was designed and piloted with a small number of adults with skeletal dysplasia. We consider this justified because of the rarity of the conditions. Considering the large number and variable presentation of skeletal dysplasias, the inclusion of only three skeletal dysplasias may be a limitation of study. However, we considered short stature, disproportion, deformities and limited joint function to be the major features affecting functioning of adults with skeletal dysplasia. The included three skeletal dysplasias all present with severe disproportionate short stature. Diastrophic dysplasia is one of the most severe skeletal dysplasias with significant deformities and joint contractures. Achondroplasia is the most common skeletal dysplasia world-wide and it was important to include this disorder in our study.

The questionnaire might be suitable for other skeletal dysplasia, but as the number of individuals in these populations is even smaller, international collaboration would be needed for greater samples to test the questionnaires suitability for all skeletal dysplasias. Nevertheless, we aimed for comprehensive evaluation of functioning based on an internationally accepted ICF core set for wider applicability. The musculoskeletal post-acute ICF core set was used as the main challenges relate to skeletal condition. The questions were not based on the diagnosis, the form of disability nor the stature of the person. They focus on the challenges and consequences on functioning that may arise in daily life for a person with disability. Moreover, there are many issues specifically in childhood, but were not included in the questionnaire, as it was designed for adults only.

## Conclusions

We developed a self-report questionnaire based on the musculoskeletal post-acute ICF core set with 15 additional ICF categories and one on chapter level to study health, functioning, disability and accessibility in people with skeletal dysplasia. The content validity was well accepted by the target group: the content was comprehensive, the operationalized items sufficiently relevant and measured the construct, and the questionnaire was feasible to use. We are confident that the developed ICF-based questionnaire can produce relevant data on life situations in people affected by skeletal dysplasia and short stature. The obtained data can be utilized to better understand their health, functioning, disability and challenges with accessibility in order to develop care and rehabilitation policies, and to plan services.

## Supplementary Information

**Additional file 1.** Number of items and item sources of the questionnaire for people with skeletal dysplasias.

## Data Availability

The anonymous data qualitative data from the thematic interviews in Finnish is not publicly available but reasonable requests to the access of data will be considered by the corresponding author. All other data generated or analysed during this study are included in this published article and its supplementary information files. The comprehensive musculoskeletal ICF core set is available at https://www.icf-research-branch.org/icf-core-sets. The PROMIS© items that were used in this study are available in English and Spanish from Health Measures service www.healthmeasures.net, but restrictions apply to the availability of other languages of these items, which were used under license for the current study, and so are not publicly available. The items are however available from the authors upon reasonable request and with permission of PROMIS Health Organization. The questionnaire is in Finnish and is available from the corresponding author on reasonable request.

## Abbreviations

A&P: Activity and participation
BF: Body functions
BS: Body structures
CHIEF: Craig hospital inventory of environmental factors
EF: Environmental factors
ICD-10: International statistical classification of diseases and related health problems 10th revision
ICD-11: International classification of diseases for mortality and morbidity statistics 11th revision
ICF: International classification for functioning, disability and health
MQE: Measure of the quality of the environment
PROMIS: Patient-reported outcomes measurement information system
WHO: World health organization
