# Supplementary material for: Towards an ICF-based self-report questionnaire for people with skeletal dysplasia to study health, functioning, disability and accessibility
Source: Orphanet J Rare Dis. 2021 May 22;16:236. doi: 10.1186/s13023-021-01857-7 (PMC8141148; doi:10.1186/s13023-021-01857-7)
Supplement: Supplementary file 1 — Additional file 1. Number of items and item sources of the questionnaire for people with skeletal dysplasias. [file 13023_2021_1857_MOESM1_ESM.docx]

# Additional Files

Table S1. Number of items and item sources of the questionnaire for people with skeletal dysplasias.

| **ICF-code*** | **ICF title** | **ICF category identified in thematic interviews** | **Number of identified items (n=522)** | **Sources of identified items (number of items)** | **Number of ICF-linked items in the questionnaire (n=173)** | **Item identification or explanation** |
| --- | --- | --- | --- | --- | --- | --- |
| **Body structures** | |  | **n=0** |  | **n=4** |  |
| s7* | Structures related to movements | Yes | 0 | .. | 3 | RG based on s7 definition and thematic interview |
| s710 | Structure of head and neck region |  | 0 | .. | 1 | RG based on diagnoses ( Table 1) |
| s720 | Structure of shoulder region |  | 0 | .. | 0 | Same as in s710 |
| s730 | Structure of upper extremity |  | 0 | .. | 0 | Same as in s711 |
| s740 | Structure of pelvic region |  | 0 | .. | 0 | Same as in s712 |
| s750 | Structure of lower extremity |  | 0 | .. | 0 | Same as in s713 |
| s760 | Structure of trunk |  | 0 | .. | 0 | Same as in s714 |
| s810 | Structure of areas of skin |  | 0 | .. | 0 | Same as in s715 |
| **Body functions** | |  | **n=294** |  | **n=55** |  |
| b130 | Energy and drive functions | Yes | 108 | PROMIS FAT (108) | 2 | An3, FATEXP40 |
| b134 | Sleep functions | Yes | 28 | PROMIS SLE (27), EDANG (1) | 4 | Sleep116, Sleep20, Sleep44, Sleep109 |
| b152 | Emotional functions |  | 71 | PROMIS EDANX(26), EDANG (26), EDDEP (19) | 3 | EDANG09, EDANX40, EDDEP04 |
| b260 | Proprioceptive function |  | 0 | .. | 1 | RG based on b260 definition |
| b270 | Sensory functions related to temperature and other stimuli |  | 0 | .. | 1 | RG based on b270 definition |
| b280 | Pain | Yes | 82 | PROMIS PainIn (3), Painbe (39), PainInt (40) | 4 | PAINQU8, PAINBE37, PAINBE8, PAININ9 |
| b415 | Blood vessel functions |  | 0 | .. | 4 | RG based on b415 definition |
| b435 | Immunological system functions | Yes | 0 | .. | 3 | RG based on b435 definition |
| b440 | Respiration functions |  | 0 | .. | 1 | RG based on b440 definition |
| b455 | Exercise tolerance functions |  | 5 | PROMIS PF(5) | 1 | PFC12 |
| b525 | Defecation functions |  | 0 | .. | 3 | RG based on b525 definition |
| b530 | Weight maintenance functions |  | 0 | .. | 1 | RG based on b530 definition |
| b620 | Urination functions |  | 0 | .. | 2 | RG based on b620 definition |
| b710 | Mobility of joint functions | Yes | 0 | .. | 9 | RG based on b710 definition |
| b715 | Stability of joint functions | Yes | 0 | .. | 9 | RG based on b715 definition |
| b730 | Muscle power functions | Yes | 0 | .. | 1 | RG based on b730 definition |
| b735 | Muscle tone functions |  | 0 | .. | 1 | RG based on b735 definition |
| b740 | Muscle endurance functions |  | 0 | .. | 0 | Same as in b730 |
| b755 | Involuntary movement reaction functions |  | 0 | .. | 1 | RG based on b755 definition |
| b760 | Control of voluntary movement functions |  | 0 | .. | 1 | RG based on b760 definition |
| b770 | Gait pattern functions |  | 0 | .. | 1 | RG based on b770 definition |
| b780 | Sensations related to muscles and movement functions |  | 0 | .. | 1 | RG based on b780 definition |
| b810 | Protective functions of the skin |  | 0 | .. | 1 | RG based on b810 definition |
| **Activity and Participation** | |  | **n=135** |  | **n=62** |  |
| d155 | Acquiring skills | Yes | 0 | .. | 1 | RG based on d155 definition |
| d177 | Making decisions |  | 0 | .. | 1 | RG based on d177 definition |
| d230 | Carrying out daily routine |  | 5 | PROMIS SRPSAT (3), EDANX (2) | 1 | SRPSAT49r1 |
| d240 | Handling stress and other psychological demands |  | 5 | PROMIS II Pos (5) | 2 | RG based on II3, II8 |
| d310 | Communicating with - receiving - spoken messages |  | 0 | .. | 1 | RG based on d310 definition |
| d410 | Changing basic body position | Yes | 14 | PROMIS PF | 4 | PFA3, PFA9, PFC45, PFC53 |
| d415 | Maintaining a body position |  | 7 | PROMIS PF | 1 | PFB42 |
| d420 | Transferring oneself |  | 1 | PROMIS PF | 1 | PFC46 |
| d430 | Lifting and carrying objects |  | 6 | PROMIS PF | 1 | PFA5 |
| d440 | Fine hand use |  | 18 | PROMIS PF | 1 | PFA22 |
| d445 | Hand and arm use | Yes | 16 | PROMIS PF | 5 | PFA29, PFA12, PF_23, RG (2 items) |
| d450 | Walking | Yes | 8 | PROMIS PF | 3 | PFB49, PFC36, PFC6 |
| d455* | Moving around | Yes | 15 | PROMIS PF | 4 | PFA1, PFC37, PFC29, RG |
| d460 | Moving around in different locations |  | 2 | PROMIS PF | 5 | PFB54, PFC56, RG (3 items) |
| d465 | Moving around using equipment |  | 1 | PROMIS PF MobAids | 1 | RG based on PF_3 |
| d470* | Using transportation | Yes | 0 | .. | 2 | RG based on d470 definition |
| d510 | Washing oneself |  | 7 | PROMIS PF | 3 | PFA55, PFB26, RG |
| d520 | Caring for body parts | Yes | 4 | PROMIS PF | 1 | PFB41 |
| d530 | Toileting | Yes | 1 | PROMIS PF | 1 | PFC51 |
| d540 | Dressing | Yes | 8 | PROMIS PF | 1 | PFA16 |
| d550 | Eating | Yes | 1 | PROMIS PF | 1 | PFA20 |
| d560 | Drinking |  | 1 | PROMIS PF | 1 | PFB29 |
| d570 | Looking after one's health |  | 1 | PROMIS II Pos | 1 | RG based on II31 |
| d620* | Acquisition of goods and services | Yes | 0 | .. | 3 | RG based on d620 definition |
| d630* | Preparing meals | Yes | 0 | .. | 1 | RG based ond630 definition |
| d640* | Doing housework | Yes | 0 | .. | 6 | SRPPER23_CaPS, SRPSAT49r1, RG based on d640 definition (4 items) |
| d750* | Informal social relationships |  | 3 | PROMIS SRPPER (2), COMP (1) | 3 | SRPPER46_CaPS, SRPSAT33_ CaPS, RG based on UCLA15x2 |
| d760 | Family relationships |  | 2 | PROMIS SRPSAT | 2 | SRPSAT15_C, SRPPER18_CaPS |
| d850* | Remunerative employment | Yes | 7 | PROMIS SRPSAT (6), SRPPER (1) | 2 | SRPSAT24r1, SRPSAT07 |
| d920* | Recreation and leisure | Yes | 1 | PROMIS SRPPER | 1 | SRPPER11_CaPS |
| d940* | Human rights | Yes | 1 | CHIEF-21 | 1 | RG based on CHIEF-21 |
| **Environmental factors** | |  | **n=93** |  | **n=52** |  |
| e110 | Products or substances for personal consumption |  | 0 | .. | 1 | RG based on e110 definition |
| e115 | Products and technology for personal use in daily living | Yes | 2 | CHIEF-10-11 | 1 | RG based on CHIEF-10-11 |
| e120 | Products and technology for personal indoor and outdoor mobility and transportation | Yes | 1 | CHIEF-10 | 3 | RG based on CHIEF-10 |
| e125 | Products and technology for communication |  | 0 | .. | 1 | RG based on e125 definition |
| e150 | Design, construction and building products and technology of buildings for public use | Yes | 4 | CHIEF-3-4 & MQE-59-62 | 1 | RG based on CHIEF-3-4 & MQE-59-62 |
| e155* | Design, construction and building products and technology of buildings for private use | Yes | 3 | CHIEF-2 & MQE-57-58 | 2 | RG based on CHIEF-2 & MQE-57- 58 |
| e225 | Climate |  | 3 | CHIEF-5 & MQE-74-75 | 1 | RG based on CHIEF-5 & MQE-74-75 |
| e245* | Time-related changes | Yes | 0 | .. | 1 | RG based on e245 definition |
| e3# | Support and relationships | Yes | 35 | PROMIS EMS (14), INF (8), INS (13) | 3 | RG based on FSE31053x2, FSE31054x2, RG based on SS6 |
| e310 | Immediate family | Yes | 2 | PROMIS EMS (2) | 1 | RG based on GS2x |
| e320 | Friends | Yes | 6 | PROMIS EMS (3), CHIEF-19 (1) & MQE-2,3 (2) | 1 | RG based on CHIEF-19 & MQE-2,3 |
| e340 | Personal care providers and personal assistants | Yes | 0 | .. | 1 | RG based on e340 definition |
| e345* | Strangers | Yes | 1 | CHIEF-20 | 1 | RG based on CHIEF |
| e355 | Health professionals |  | 0 | .. | 0 | Included in e3 |
| e4# | Attitudes | Yes | 4 | PROMIS ISO (4) | 1 | Iso-CaPS2 |
| e410 | Individual attitudes of immediate family members |  | 0 | .. | 0 | RG based on e410 definition |
| e420 | Individual attitudes of friends |  | 0 | .. | 1 | RG based on e420 definition |
| e430 | Individual attitudes of people in positions of authority |  | 0 | .. | 0 | Included in e4 |
| e440 | Individual attitudes of personal care providers and personal assistants |  | 0 | .. | 0 | Included in e4 |
| e445* | Individual attitudes of strangers | Yes | 2 | CHIEF-17 & MQE-12 | 1 | RG based on CHIEF-17 & MQE-12 |
| e450 | Individual attitudes of health professionals | Yes | 1 | MQE-11 | 1 | RG based on MQE-11 |
| e455* | Individual attitudes of other professionals | Yes | 1 | MQE-11 | 1 | RG based on MQE-11 |
| e460* | Societal attitudes | Yes | 1 | MQE-13 | 1 | RG based MQE-13 |
| e555 | Associations and organizational services, systems and policies |  | 1 | Finsote basic survey for 20-54 years, item 17 | 1 | RG based on Finsote item 17 |
| e575 | General social support services, systems and policies | Yes | 26 | Finsote basic survey for 20-54 years, item 81 | 27 | RG based on Finsote item 81 |
| e580 | Health services, systems and policies | Yes | 0 | Same as for e757 | 0 | Included in e575 |

*Added ICF category to the musculoskeletal post-acute ICF core set based on linking transcriptions from the thematic interviews to the ICF categories. #Added chapter level ICF category based on item operationalisation. Abbreviations: CHIEF=Craig Hospital Inventory of Environmental Factors, COMP=PROMIS v2.0 Companionship–Short Form 6a, EDANG=PROMIS Item Bank v1.1 Emotional Distress-Anger, EDANX=PROMIS Item Bank v1.0 Emotional Distress-Anxiety–Calibrated Items, EDDEP=PROMIS Item Bank v1.0 Emotional Distress-Depression–Calibrated Items, EMS=PROMIS v2.0 Emotional support–Short Form 8a, FAT=PROMIS Item Bank v1.0 Fatigue–Calibrated Items, II Pos=PROMIS Item Bank v1.0 Illness Impact Positive, INF=PROMIS v2.0 Informational Support–Short Form 8a, INS=PROMIS v2.0 Instrumental Support–Short Form 8a, ISO=PROMIS Item bank v2.0 Social Isolation–Short Form 8a, MQE=Measure of the Quality of the Environment, Painbe=PROMIS Item Bank v1.1Pain behavior–Calibrated Items, PainIn=PROMIS Item Bank v1.0 Pain intensity–Scale, PainInt=PROMIS Item Bank v1.1 Pain Interference, PF=PROMIS Item Bank v1.2 Physical function, PF MobAid=PROMIS Item Bank v1.0–Physical Function with Mobility, Sleep=PROMIS Item Bank v1.0 Sleep Disturbance–Calibrated Items, SRPPER=PROMIS Item Bank v2.0 Ability to Participate in Social Roles and Activities–Short Form 8a, SRPSAT=PROMIS Item Bank v1.0 Satisfaction with Participation in Social Roles–Calibrated Items.
